# Use of Circular RNAs in Diagnosis, Prognosis and Therapeutics of Renal Cell Carcinoma

**DOI:** 10.3389/fcell.2022.879814

**Published:** 2022-06-22

**Authors:** Rebeca Osca-Verdegal, Jesús Beltrán-García, José Luis Górriz, José María Martínez Jabaloyas, Federico V. Pallardó, José Luis García-Giménez

**Affiliations:** ^1^ Center for Biomedical Network Research on Rare Diseases (CIBERER), Institute of Health Carlos III, Valencia, Spain; ^2^ Department of Physiology, Faculty of Medicine and Dentistry, University of Valencia, Valencia, Spain; ^3^ INCLIVA Biomedical Research Institute, Valencia, Spain; ^4^ Department of Nephrology, University Clinic Hospital, INCLIVA, University of Valencia, Valencia, Spain; ^5^ Department of Urology, University Clinic Hospital, INCLIVA, University of Valencia, Valencia, Spain; ^6^ EpiDisease S.L. (Spin-Off CIBER-ISCIII), Parc Científic de la Universitat de València, Valencia, Spain

**Keywords:** RCC (Renal Cell Carcinoma), circRNAs, biomarker, diagnosis, therapy, cancer

## Abstract

Renal cell carcinoma is the most common type of kidney cancer, representing 90% of kidney cancer diagnoses, and the deadliest urological cancer. While the incidence and mortality rates by renal cell carcinoma are higher in men compared to women, in both sexes the clinical characteristics are the same, and usually unspecific, thereby hindering and delaying the diagnostic process and increasing the metastatic potential. Regarding treatment, surgical resection remains the main therapeutic strategy. However, even after radical nephrectomy, metastasis may still occur in some patients, with most metastatic renal cell carcinomas being resistant to chemotherapy and radiotherapy. Therefore, the identification of new biomarkers to help clinicians in the early detection, and treatment of renal cell carcinoma is essential. In this review, we describe circRNAs related to renal cell carcinoma processes reported to date and propose the use of some in therapeutic strategies for renal cell carcinoma treatment.

## Introduction

### General Concepts

Renal cell carcinoma (RCC) originates in the lining of the proximal convoluted tubule making up part of the very small tubes in the kidney and contributing to the formation of primary urine by the nephron.

**FIGURE 1:**
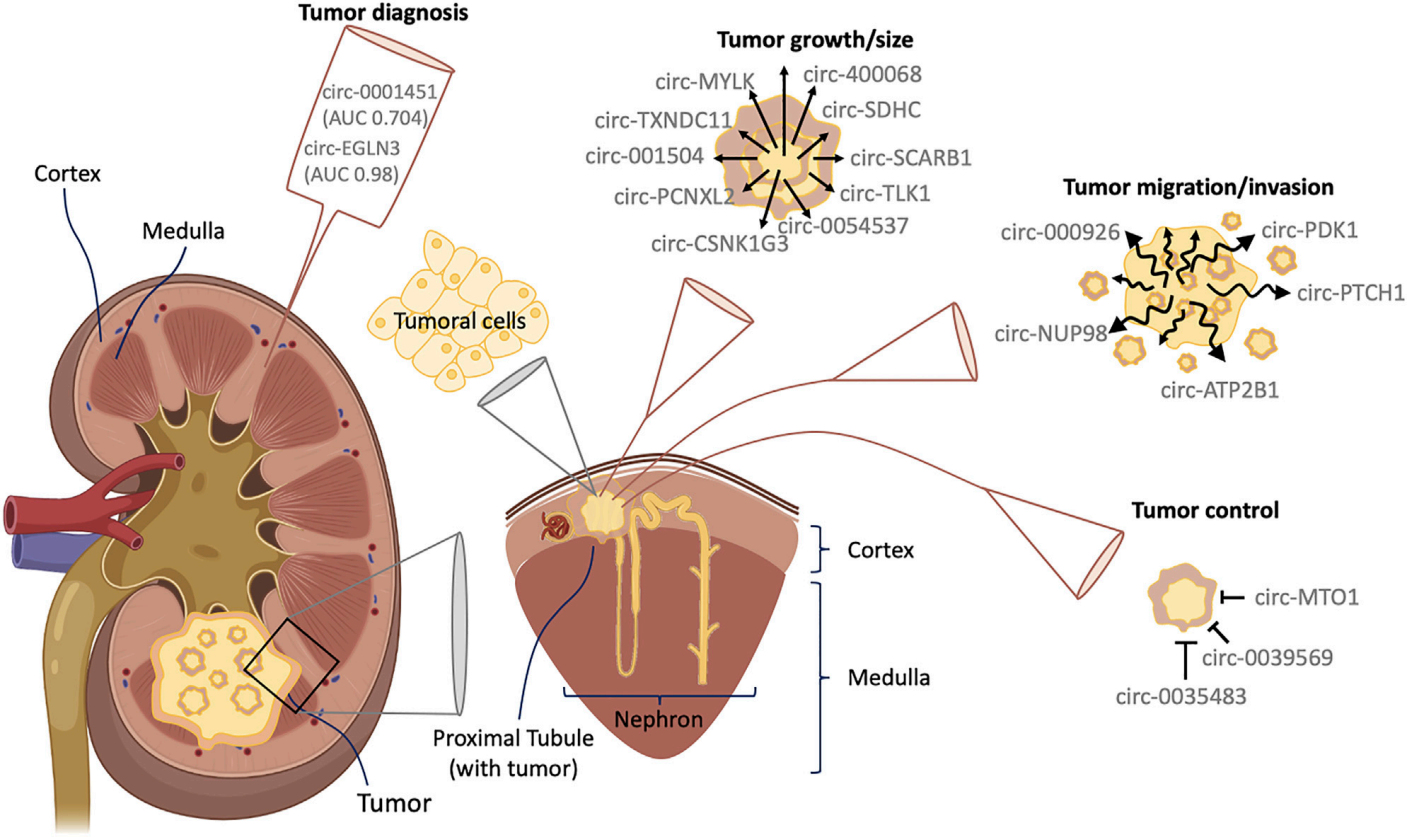
Interaction between circRNAs and RCC. RCC comes from epithelial cells in the proximal tubule, located in the renal cortex of the nephron. The figure shows some interactions described in the main text between circRNAs and RCC as well as those proposed as good candidates for RCC diagnosis.

In adults, RCC is the most common type of kidney cancer, representing up to 90% of kidney cancer diagnoses, with at least 70% of cases being clear cell renal cell carcinoma (ccRCC), which represents most cases and produces the greatest number of deaths. It is identified because, in this tissue, cells appear under the microscope as clear or pale (Tretiakova, 2020). Other subtypes of RCC, with different histology and development degree, are papillary. Rare subtypes for RCC have been also identified such as Chromophobe and collecting duct RCC (Delahunt et al., 2007). Clear cells can range from grade 1 (slow tumor growth type) to grade 4 (fast tumor growth type) which is commonly associated with metastasis (Karumanchi et al., 2002; DeVita et al., 2008). Likewise, there is a wide variety of specialized cells located throughout the nephron that can play a relevant role in the development of RCC, which is composed of several types of histological cells (reviewed in-depth by Tretiakova (2020)). In fact, it has been demonstrated that both papillary RCC and ccRCC arise from the epithelium of the proximal tubule (Cairns, 2011) (Figure 1). Notably, the incidence rate of RCC has increased 2%–4% annually in the last years, having doubled with no clear explanation in the last 25 years (Motzer et al., 2009; Cairns, 2011). Interestingly, despite the incidence having doubled from 1975 to 2016, the age-standardized mortality remains the same (Padala et al., 2020).

Between 2% and 4% of RCC are hereditary (Menko and Maher, 2016) and are sometimes inherited in an autosomal dominant manner. Therefore, genetic screening is recommended for identifying high-risk patients with hereditary RCC, especially when ≥2 relatives are affected in the absence of syndromic features (Maher, 2018). It is known that individuals with advanced stage of chronic kidney disease undergoing long-term dialysis also have a high risk of RCC (Cairns, 2011). Likewise, the most common hereditary RCC appears in patients with a mutation in the *VHL* gene (located in chromosome 3p), causing the von Hippel-Lindau disease (VHL) (ORPHA:892), with a predisposition to present ccRCC, due to the role of the *VHL* gene as a tumor suppressor. Patients presenting VHL syndrome show many cysts in the kidney and multiple bilateral ccRCC. This is due, in part, to over expression of both insulin-like growth factor 1 (IGF-1), that causes dysregulated cell growth, and hypoxia-inducible factors (HIF), which in turn help to generate vascular endothelial growth factor (VEGF), leading new blood vessels to grow and finally form cysts and tumors in the kidney (Latif et al., 1993; Pavlovich et al., 2003; DeVita et al., 2008). When RCC is detected in young patients, it usually indicates VHL disease (Motzer et al., 2009).

RCC tumorigenesis is an extremely complex process, that involves both genetic and epigenetic alterations. However, nowadays, only a few types of RCCs can be explained by genetic predisposition (Valladares Ayerbes et al., 2008). Also, epigenetic alterations in RCCs are still poorly understood. Recently, Li R.-G. et al. (2021) reported that histone methyltransferase G9a promotes RCC development by methylation of H3K9me2, thus silencing the tumor suppressor gene serine peptidase inhibitor Kazal-type 5 (*SPINK5*). In fact, although genetic factors are highly related to the development of cancer, environmental and lifestyle factors are strongly involved in the onset of RCC (Leppert and Patel, 2016).

Regarding current treatments for RCC, surgical resection remains the main therapeutic strategy. Nonetheless, local or distant metastasis may still occur in some patients, even after radical nephrectomy, and the majority of RCCs are resistant to chemotherapy and radiotherapy once metastasis appears, thereby highlighting the need of developing new therapies aimed at improving patient outcomes (Pal and Agarwal, 2016; Lieder et al., 2017; Lara and Evans, 2019).

The new era of modern therapeutic options for RCC was initiated in 2006, with the approval of sunitinib, a pioneer tyrosine kinase inhibitor (TKI) targeting VEGF to avoid the metastatic state in RCC (mRCC). Patients with mRCC who were treated with sunitinib showed significantly prolonged overall survival after several years of stagnation. From this point, many other TKIs have been developed against different targets (e.g., VEGF, MET, PDGFR) and mTOR inhibitors, that demonstrated improved survival in other lines of therapy (Hsieh et al., 2017; Santoni et al., 2018). Recently, efforts have been directed to the development of the immune checkpoint inhibitors (ICI), which has significantly improved the current therapeutic options for RCC, when complete remission of metastasis was observed. In this regard, multiple ICIs are used in different therapeutic lines, but, most mRCC patients did not demonstrate durable clinical benefits, and it is common to find primary resistance to these therapies or acquire resistance after an initial response. Likewise, the combination of both modalities was recently approved for first-line mRCC treatment (Stühler et al., 2021). Specifically, the combination of pembrolizumab with axitinib was included in the updated European Association of Urology guideline recommendations for first-line treatment of mRCC in 2019, based on the phase 3 KEYNOTE-426 trial, that demonstrated the superiority of this therapy over sunitinib in overall response rate (RR), progression-free survival (PFS) and overall survival (OS) (Albiges et al., 2019; Motzer et al., 2019a; Rini et al., 2019). In May 2019, the US Food and Drug Administration (FDA) approved another ICI-TKI combination of avelumab with axitinib for first-line treatment in mRCC patients, based on improved PFS results from the phase 3 JAVELIN Renal 101 trial (Motzer et al., 2019b). The combination of nivolumab with cabozantinib has recently been approved by the FDA for first-line treatment of mRCC patients in January 2021, based on the positive ORR, PFS, and OS data from the CheckMate 9ER trial (Choueiri et al., 2020). The recent results obtained with the combination of both therapeutic strategies, raise the question of whether the combination of ICI with TKI can set the basis for the new standard therapy for mRCC patients. However, new therapeutic approaches as well as new biomarkers, able to perform a precise patient selection based on molecular responses, will help in the choice of first-line therapy between TKI, ICI, or both, leading to better outcomes in the respective subgroups and avoiding unnecessary toxicity.

The diagnosis of RCC remains challenging, mainly because kidney cancer is usually clinically silent. Indeed, RCC is often diagnosed when it has already metastasized to other organs (i.e., 45% to lung, 30% to bone, 22% to lymph node, 20% to liver, 8% to brain) (Brufau et al., 2013). Moreover, when early clinical manifestations are presented, they are usually diverse and unspecific, and are often misattributed, due to their heterogeneity. In this regard, when patients show the classic triad of hematuria, pain and a flank mass, they are usually at an advanced disease stage. Nevertheless, more than 40% of patients with RCC do not present any of these clinical symptoms (Gibbons et al., 1976), and actually, about 60% of patients with this type of cancer are detected incidentally in early stages (Oda et al., 2001). In this regard, some authors such as Cairns et al. have proposed that RCC is the genitourinary cancer with the highest mortality rate (Cairns, 2011; Turajlic et al., 2018). In addition to the heterogeneity of this type of cancer, its wide range of clinical manifestation are of note, including from neoplasms with no metastasis potential to rapidly progressive tumors (Frew and Moch, 2015).

### Circular RNAs Characterization and Analysis

circRNAs are exceptional biomarkers thanks to the lack of free ends, which make them more stable than their linear counterparts. Additionally, the structural conformation (covalently closed loop that lacks free 3′ and 5′ ends) of circRNAs makes them very stable in blood (Memczak et al., 2015) and resistant to exonucleases. Importantly, circRNAs show an average half-life of about 48 h compared to the 10 h of linear RNAs in plasma (Memczak et al., 2013; Jeck and Sharpless, 2014). The biogenesis process of circRNAs is different from the canonical splicing that forms mRNAs. circRNAs are produced by back-splicing process mediated by a spliceosome, being able to contain the transcription of protein-coding gene’ exons (Bolha et al., 2017), or introns (Jeck and Sharpless, 2014; Zheng et al., 2016). Moreover, the circular form of those RNAs is thanks to the link that is produced between the upstream acceptor splice site (3′ splice site) and the downstream splice donor site (5′ splice site) on the same exon or others (Salzman et al., 2012; Memczak et al., 2013; Ashwal-Fluss et al., 2014; Zhang et al., 2014; Chen and Yang, 2015; Wang and Wang, 2015; Diallo et al., 2019).

Current analytical tools to investigate circRNA expression and sequence are based on the current methods we use for the analysis of other genetic material. In this regard, arrays are the most used technique for circRNA analysis, although the analysis is limited to current known circRNAs of which probes have been printed onto the array. This limitation has prompted the development of alternative strategies to discover new circRNAs by high-throughput circRNA sequencing (Circ-seq), which allows to perform the differential expression analysis and the identification of thousands of circRNAs from an initial sample of mixed RNAs. The sample can be processed to enrich circRNAs by digesting the initial sample with RNase R to eliminate most linear transcripts while circRNAs remains intact, due to they are resistant to exonucleases (Jeck et al., 2013; Salzman et al., 2013). Nevertheless, circRNAs enrichment is not always necessary if a high number of lectures can be performed when samples are sequenced (Pandey et al., 2020). Other technique used for circRNA detection and quantification is PCR, both RT-qPCR (reverse transcription followed by quantitative polymerase chain reaction) or dd-PCR (droplet digital PCR). ddPCR is more precise, reproducible, provides absolute numbers, and measures low-abundance RNAs than RT-qPCR (Taylor et al., 2017; Pandey et al., 2020) and it is further recommended for absolute quantification and fluid circulating circRNAs analysis.

It is noteworthy, the bioinformatic analysis is essential to discover new circRNAs. However, circRNA annotations and analysis algorithms are still in development. In any case, bioinformatic protocols are also essential to understand the role of circRNAs controlling specific biological pathways involved in cell differentiation, progression, etc. In this regard, new bioinformatic procedurs are aimed to predict if a circRNA has or not the ability of binding to a protein and predict if a circRNA is able to interact with more than one microRNA (Dori and Bicciato, 2019).

### Potential Use of Circular RNA in Clinical Settings

RCC diagnosis and prognosis is a current medical need. Many biomarkers have been proposed in recent years, including circular RNAs (circRNAs), which are considered a hot research topic (Beltrán-García et al., 2020). circRNAs are associated with the onset, development, and progression of a wide range of diseases (Zhang et al., 2018), including immune diseases, cardiovascular and neurological diseases, and many types of cancer (Floris et al., 2017; Li et al., 2018; Zhong et al., 2018). The main reasons for the high popularity of circRNAs are that these non-coding RNAs are directly involved in gene regulation, have demonstrated high sequence conservation among mammalian organisms, have shown to be present in different biospecimens (i.e. showing high stability in biofluids, including plasma), and are usually more abundant than other class of regulatory RNAs, because exonucleases are not able to degrade circRNAs (Beltrán-García et al., 2020). These properties postulate circRNAs as promising molecules for clinical settings such as diagnosis, prognosis and therapeutics. Moreover, because of their ability to indicate specific disease development stages and because they are tissue-specific (Memczak et al., 2013), circRNAs may be good biomarkers for the stratification of different diseases, including many cancers.

This review describes the role of this new class of regulatory RNAs in RCC and the feasibility of using circRNAs as diagnostic and prognosis biomarkers, as well as the enormous possibilities to develop new therapeutic strategies based on the use of circRNAs, thereby opening up new avenues for personalized circRNA-based medicine.

## Circular RNAs in Renal Cell Carcinoma

CircRNAs promote the development and progression of RCC usually through circRNA-miRNA-mRNA interaction networks. However, this regulation, which is sometimes mediated by circRNAs, is due to their direct binding to proteins, modulating their availability and function (Wang et al., 2020). In this regard, some authors have performed genome-wide transcriptional analysis to clarify the different profiles of circRNAs that are involved in RCC. Ma et al. (2019) found 542 circRNAs abnormally expressed in ccRCC using a pre-designed circRNA microarray. Among these, 324 circRNAs were significantly downregulated, while 218 were upregulated in ccRCC tumors. Likewise, Franz et al. found 13,261 circRNAs differentially expressed in RCC using the Arraystar circRNA microarray (Arraystar Inc.; Rockville, MD, United States). The authors found that 78 circRNAs were upregulated and 91 were downregulated more than two-fold (Franz et al., 2019).

Other authors, using bioinformatic analysis, explored the expression profile of circRNAs in RCC. Jiang et al. used different bioinformatic methods and integrated analysis of a competing endogenous RNA network in RCC and found six differentially expressed circRNAs. Of these six circRNAs, three were predicted to be upregulated (hsa_circ_0031594, hsa_circ_0029340, and hsa_circ_0039238) and the other three were downregulated (hsa_circ_0084927, hsa_circ_0035442, and hsa_circ_0025135) (Jiang and Ye, 2019).

The different high-throughput results obtained in the studies mentioned above, strongly postulate the potential of circRNAs as potential feasible clinical biomarkers for cancer diagnosis, specifically in the development and pathogenesis of RCC. However, its molecular mechanisms and its specific role in RCC remain unknown, and further studies are required to clarify their specific role in RCC.

### Circular RNAs Implicated in Kidney Cancer Proliferation

Many circRNAs have been described to be related to cell growth and cancer proliferation both *in vitro* and *in vivo* (Wang et al., 2020) (Figure 1). circRNAs play a fundamental role in the regulation of expression of many genes and proteins (Beltrán-García et al., 2020), with a high number being cancer proliferation-related genes. In this section we describe circRNAs related to RCC proliferation pathways, which have already been validated (Table 1).

**TABLE 1:** circRNAs described in different processes related to cancer development.

circ-RNA	miRNA regulated	Gene regulated	Process regulated	Clinical utility	Biospecimen	Ref
**↑**circ-0054537	miR-130a-3p	*cMET*	Proliferation and tumorigenesis	RCC biomarker	RCC tissue	Li R. et al. (2020)
**↑**circ-SCARB1	miR-510-5p	*SDC3*	Proliferation and tumorigenesis	RCC biomarker	RCC tissue	Sun et al. (2020a)
**↑**circ-MTO1	miR-9	*LMX1A*	Inhibits proliferation and invasion	RCC diagnosis and therapy	Renal cancer cells and cell culture	Li K. et al. (2020)
	miR-223					
**↑**circ-EGLN3 (AUC 0.98)	miR-1299	*IRF7*	Proliferation and aggressiveness	RCC diagnosis and therapy	RCC tissue	Franz et al. (2019); Lin and Cai, (2020); Wang et al. (2020); Zhang et al. (2021)
	miR-1224-3p	*HMGXB3*	Proliferation, migration and invasion			
**↑**circ-0039569	miR-34a-5p	*CCL22*	Inhibits proliferation and invasion	RCC diagnosis and therapy	RCC tissue and cells	Jin et al. (2019)
**↑**circ-0001451 (AUC 0.704)	N.A	N.A	Proliferation and development	RCC diagnosis and therapy	ccRCC cells and cell culture	Wang et al. (2018)
**↓**circ-RAPGEF5	miR-27a-3p	*TXNIP*	Proliferation and migration	RCC diagnosis and therapy	RCC tissue and cell culture	Chen et al. (2020)
**↑**circ-PCNXL2	miR-153	*ZEB2*	Proliferation, tumor growth and invasion	RCC diagnosis and therapy	ccRCC tissue and cell culture	Zhou et al. (2018)
**↑**circ-SDHC	miR-127-3p	*CDKN3*	Proliferation, migration and poor survival	RCC stage and therapy	RCC tissue	Cen et al. (2021)
**↑**circ-PDK1	miR-377-3p	*NOTCH1*	Tumor metastasis and renal cell invasion	RCC diagnosis and therapy	RCC tissue	Huang et al. (2020)
**↑**circ-TXNDC11	N.A	N.A	Tumor growth and invasion	RCC stage and therapy	Cell culture and mice model	Yang et al. (2021)
**↑**circ-001287	miR-144	*CEP55*	Proliferation, tumor growth, migration, and invasion	RCC diagnosis and therapy	RCC tissue and cells	Feng et al. (2020)
**↑**circ-001504	miR-149	*NUCB2*	Tumor growth	RCC diagnosis and therapy	RCC tissue and cells	Xin et al. (2021)
**↓**circ-UBAP2	miR-148a-3p	*FOXK2*	Proliferation, migration, and invasion	RCC diagnosis and therapy	ccRCC tissue and cell culture	Sun et al. (2020b)
**↑**circ-PTCH1	miRNA-485-5p	*MMP14*	Migration, invasion and metastases	RCC diagnosis	RCC tissue	Liu et al. (2020)
**↑**circ-CSNK1G3	miR-181b	*TIMP3*, *LATS2*, *NDRG2* and *CYLD*	Tumor growth and metastases	RCC diagnosis and therapy	RCC tissue	Li W. et al. (2021)
**↑**circ-000926	miR-411	*CDH2*	Growth, migration, and invasion	RCC diagnosis and therapy	RCC tissue	Zhang et al. (2019)
**↑**circ-0035483	miR-31-5p	*HMGA1*	Inhibits the malignant RCC cells behavior	RCC diagnosis and therapy	RCC tissue and cells	Liu et al. (2021)
**↑**circ-001842	miR-502-5p	*SLC39A14*	Poor prognosis, tumor growth, cell proliferation, migration and invasion	RCC diagnosis, prognosis and therapy	RCC tissue and cell culture	Li et al. (2020b)
**↑**circ-MYLK	miR-513a-5p	*VEGF-C*	Tumor size, distant metastasis and poor prognosis	RCC diagnosis and therapy	RCC cells	Li et al. (2020b)
**↑**circ-PRRC2A	miR-514a-5p	*TRPM3*	and worse survivorship in RCC patients	RCC diagnosis, prognosis and therapy	RCC tissue and cell culture	Li W. et al. (2020)
	miR-6776-5p					
**↑**circ-TLK1	miR-495-3p	*CBL*	Proliferation, migration and invasion, thus facilitating proliferation and metastasis	RCC diagnosis and therapy	RCC tissue and cells	Li et al. (2020a); Lei et al. (2021)
	miR-136-5p	*CBX4*	Proliferation and metastasis, and correlates with *VEGF-A*			
**↓**circ-EXOC7	miR-149-3p	*CSF1*	Initiation and progression linked to AR	Sex-specific RCC diagnosis and prognostic biomarkers and therapy	RCC tissue and cell culture	Gong et al. (2021)
**↓**circ-ATP2B1	N.A	*ATP2B1*	Cell invasion	Sex-specific RCC diagnosis biomarker	RCC tissue and cell culture	Han et al. (2018)
**↑**circ-SAR1A	miR-382	*YBX1*	Tumor growth and invasion	RCC diagnosis and therapy	RCC tissue	Chen et al. (2017); Zhao et al. (2020)
**↑**circ-NUP98	miR-567	*PRDX3*	Migration and invasion	Biomarker of RCC stage	RCC tissue and cell culture	Yu et al. (2020)
**↑**circ-400068	miR-210-5p	*SOCS1*	Proliferation and apoptosis	RCC diagnosis and therapy	Plasma exosomes, RCC tissue and cells	Xiao and Shi, (2020)
**↑**circ-0035483	miR-335	*CCNB1*	Increase autophagy-related proteins	RCC theragnosis biomarker and therapy	RCC tissue and cells	Yan et al. (2019)

Note: N.A, not available; circ-RNA, circular RNA; RCC, renal cell carcinoma.

Rui et al. demonstrated that certain circRNAs such as hsa_circ-0054537, can modulate tumorigenesis in RCC by binding to miR-130a-3p in a competitive way, modulating the expression of the *cMET* oncogene. The inhibition of miR-130a-3p by circRNA hsa_circ-0054537 induces overexpression of *cMET*, which, in turn, results in proliferation and tumorigenesis during the development of kidney cancer. These findings, together with the abnormally high levels of hsa_circ-0054537 found in RCC tissue and their inverse correlation with miR-130a-3p (Wotschofsky et al., 2012; Li R. et al., 2020), suggests that this circRNA may be a good candidate for use as a RCC diagnostic biomarker.

circ-SCARB1 has been found to be elevated in RCC tissue. Mechanistically, circ-SCARB1 works by upregulating *SDC3* gene expression through the inhibition of miR-510-5p. In fact, Sun *et al,* observed an inhibition of cell proliferation, migration and invasion, and an increase of apoptosis, when they produced a knockdown of circ-SCARB1 in both A497 and 786-O renal cancer cells. Thus, an anti-circ-SCARB1 could be useful as a therapeutic agent (Sun et al., 2020a). Conversely to circ-SCARB1, the circRNA circ-MTO1 plays a beneficial role in RCC because of its ability to suppress RCC cancer cell progression *via* the miR9/*LMX1A* axis. In fact, circ-MTO1 overexpression suppresses cell proliferation and metastases in both A497 and 786-O renal cancer cells, while silencing circ-MTO1 promotes proliferation of SN12C and OS-RC-2 renal cancer cells. Mechanistically, circ-MTO1 can sponge miR-9 and miR-223, leading to an increase of *LMX1A* expression, and thereby inhibiting RCC, cell proliferation, and invasion (Li K. et al., 2020).

circ-EGLN3 has been proposed as a circRNA involved in RCC proliferation and aggressiveness. In this regard, circ-EGLN3 may be a good diagnostic biomarker since elevated levels of circ-EGLN3 were found in RCC tissues, in comparison with adjacent non-carcinogenic tissue (Franz et al., 2019; Lin and Cai, 2020). In addition, this circRNA discriminated healthy from malignant tissue with an accuracy of 97% (Franz et al., 2019). Interestingly, circ-EGLN3 also demonstrated that it can predict unfavorable prognosis in RCC patients, with an area under the curve (AUC) of 0.98 (Wang et al., 2020). Regarding its molecular mechanism, circ-EGLN3 sponges miR-1299 and, therefore, enhances *IRF7* expression levels. In this regard, it was previously described that the elevation of *IRF7* levels, as a consequence of miR-1299 inhibition, is responsible for RCC cell proliferation and aggressiveness. Recently, the role of circ-EGLN3 in regulating the miR-1224-3p/HMGXB3 axis was determined, showing that it is able to sponge miR-1224-3p, which in turn regulates *HMGXB3* expression. This circRNA should be further investigated since it demonstrated a good AUC value for RCC prognosis and could therefore, be useful in the diagnosis of RCC. Furthermore, due to silencing circ-EGLN3 increases the expression of miR-1299 and miR-1224-3p, and thus decreases RCC cell proliferation, migration, and invasion (Lin and Cai, 2020; Zhang et al., 2021), making it possibly useful as a therapeutic target for RCC treatment.

Similarly, Jin et al. (2019) studied the expression of a wide range of circRNAs in RCC patients compared to healthy people. The study found 35 significantly overexpressed circRNAs, and proposed circ-0039569 as one of the most interesting. In fact, circ-0039569 was upregulated in RCC cells and tissues from patients. Likewise, the authors found that decreased levels of circ-0039569 suppressed the proliferation and metastasis of RCC cells, suggesting that this circRNA may be used as a diagnostic and prognostic biomarker in RCC. Regarding its molecular mechanism, circ-0039569 binds competitively to miR-34a-5p, which in turn induces an increase in the expression levels of the *CCL22* gene, which plays a relevant role in tumor immunity, specifically by recruiting regulatory T cells (Tregs) to the tumor, and by promoting the formation of dendritic cells-treg (Röhrle et al., 2020). Here it is important to mention that a therapy aimed at increasing levels of circ-0039569 may result in a promising strategy for the treatment of RCC.

Another circRNA that can help to diagnose ccRCC as well as being a novel target for its treatment is circ-0001451, (Wang et al., 2018). Wang et al. described the role of this circRNA in the proliferation and development of ccRCC cells, reporting that a knockdown of circ-0001451 promotes tumor growth *in vitro*. Furthermore, circ-0001451 demonstrated an AUC of 0.704 for tumor diagnosis (*p*-value of 0.006), as well as for tumor growth stage. The authors observed that the downregulation of circ-0001451 was linked with aggressive features of the tumor (*p*-value of 0.025). In addition, they also found a negative correlation with the downregulation of circ-0001451 and patient survival (*p*-value of 0.000).

Likewise, another circRNA, RAPGEF5 (circ-RAPGEF5) was significantly downregulated in RCC tissues, and lower levels of circ-RAPGEF5 demonstrated a positive correlation with aggressive clinical phenotypes (*p*-value of 0.037), poor survival (*p*-value of 0.001), and recurrence-free survival. Moreover, functional assays showed that circ-RAPGEF5 suppresses RCC proliferation and migration *in vitro* and *in vivo*. Mechanistically, circ-RAPGEF5 works by sponging the oncogenic miR-27a-3p, which, in turn, inhibits the suppressor gene *TXNIP* (Chen et al., 2020). Consequently, circ-RAPGEF5 plays an important role in suppressing RCC via the miR-27a-3p/*TXNIP* pathway.

Another interesting circRNA is circ-PCNXL2. Circ-PCNXL2 has been found to be significantly upregulated in ccRCC by circRNA microarray screening. Likewise, circ-PCNXL2 was correlated with poor overall survival of ccRCC patients. Furthermore, the inhibition of circ-PCNXL2 through a knockdown process, reduced RCC cell proliferation and invasion *in vitro*, and decreased tumor growth *in vivo* (Zhou et al., 2018). Therefore, it is also a good candidate to develop anti-circRNA therapies directed to circ-PCNXL2 inhibition. Regarding the molecular mechanism, circ-PCNXL2 sponges miR-153 and regulates *ZEB2* expression, a transcription factor involved in the signaling pathway of transforming growth factor β (TGFβ), which is essential for RCC progression and tumorigenesis.

### Circular RNAs Implicated in Kidney Cancer Migration and Invasion

RCC is prone to developing metastasis, due to its intrinsic properties to migrate and invade other tissues, as well as its inherent resistance to radiotherapy and chemotherapy (Pal and Agarwal, 2016; Lieder et al., 2017; Lara and Evans, 2019). In this regard, many circRNAs are related to the regulation of genes involved in cell migration, invasion and metastasis (Figure 1; Table 1).

In RCC patients with advanced tumor, node, and metastasis (TNM) stage and poor survival, high expression of circ-SDHC has been found. This circRNA is related, both *in vitro* and *in vivo*, to tumor cell proliferation and invasion, and progression of malignancy. Circ-SDHC binds competitively to miR-127-3p and enhances *CDKN3* expression thereby activating the E2F1 pathway. In addition, Cen et al. (2021) observed that circ-SDHC knockdown caused a decrease in *CDKN3* expression and E2F1 pathway inhibition through the increment of miR-127-3p, thus demonstrating the important implication of this circRNA in RCC. Considering the essential role of the axis circ-SDHC/miR-127-3p/CDKN3/E2F1 in RCC progression, measuring the levels of circ-SDHC may contribute to providing clinicians with information about disease stage. It has therefore been hypothesized that a therapy based on blocking circ-SDHC may be useful to avoid disease progression, thus improving the clinical phenotype.

Similarly, Huang et al. found upregulation of circ-PDK1 in RCC tissues, which demonstrated a positive correlation between circ-PDK1, tumor metastasis and renal cell invasion (*p*-value of 0.02). Regarding the molecular mechanism, they also showed that circ-PDK1 acts by sponging miR-377-3p, thus regulating *NOTCH1* expression, and finally controlling RCC invasion and metastasis. In this regard, the authors reported that the *NOTCH1* gene was able to reverse the metastasis inhibition effect after circ-PDK1 knockdown (Huang et al., 2020).

Recently, Yang et al. demonstrated the implication of circ-TXNDC11 in MAPK/ERK pathway regulation, promoting RCC growth and invasion. They observed that circ-TXNDC11 overexpression was correlated with an advanced TNM stage and lymph node metastasis of RCC. Furthermore, they proposed circ-TXNDC11 as a potential therapeutic agent for RCC treatment, since circ-TXNDC11 silencing causes cell proliferation and invasion suppression *in vitro*, and reduced tumor growth in a mice model (Yang et al., 2021).

Similarly, Feng et al. demonstrated overexpression of circ-001287 in RCC tissues in comparison with non-affected tissue. They also observed increased proliferative, tumor growth, migratory and invasiveness capacities by the circ-001287/miR-144/*CEP55* axis. Furthermore, to demonstrate the implication of circ-001287 downregulation, the authors silenced circ-001287 and observed the overexpression of miR-144 and subsequent downregulation of *CEP55,* which is implicated in kidney development and cell mitosis and cytokinesis (Fabbro et al., 2005). When this occurred, using a nude mice model, they found the inhibition of RCC cell tumorigenicity *in vivo*, and proposed circ-001287 as a new therapeutic target for RCC patients (Feng et al., 2020).

Nucleobindin 2 (*NUCB2*) codifies for a calcium-binding protein, and its overexpression has been related to tumor growth, cell proliferation, migration and invasion in prostate cancer, bladder cancer and RCC (Zhang et al., 2013; Liu et al., 2018; Xin et al., 2021). In this regard, Zhang et al. (2013) proposed this gene as a prognostic biomarker for prostate cancer, and years later Liu et al. (2018) proposed *NUCB2* as a potent prognostic biomarker in bladder cancer. Recently, Xin et al. described its implication in RCC metastasis, being regulated by circ-001504, which has been found to be upregulated in RCC. Molecularly, circ-001504 acts by increasing *NUCB2* expression competing with the endogenous miR-149. Interestingly, using a mouse model it was found that when circ-001504 was silenced, tumor growth was suppressed, through a mechanism involving *NUCB2* downregulation (Xin et al., 2021).

In the same way, another circRNA, circ-UBAP2, was found to be significantly downregulated in ccRCC tissues and derived cell lines. In fact, Sun et al. demonstrated that the overexpression of circ-UBAP2 inhibited the proliferation, migration, and invasion of ccRCC cells. Regarding the molecular mechanisms, it was found that circ-UBAP sponges miR-148a-3p, which in turn regulates the expression levels of the *FOXK2* gene (Sun et al., 2020b). Therefore, in brief, circ-UBAP2 works as a tumor suppressor in ccRCC through regulation of the miR-148a-3p/*FOXK2* axis.

### Circular RNAs Implicated in Kidney Cancer Epithelial to Mesenchymal Transition and Vascular Endothelial Growth Factor Activation

One of the most relevant signaling pathways that contributes to tumor initiation, migration and invasion is the activation of the epithelial to mesenchymal transition (EMT) process and the VEGF signaling pathway (Li W. et al., 2021). During the EMT process, epithelial cells are altered at a molecular level, changing their epithelial phenotype and acquiring mesenchymal features, thus serving as an indicator of tumor invasion and metastasis (Yeung and Yang, 2017). Regarding VEGF, it is widely accepted that blood vessels are critical for the growth of tumors thanks to the formation of neo-vessels (Folkman et al., 1963; Sherwood et al., 1971; Senger et al., 1983; Ferrara, 2002; Ferrara et al., 2004) (Table 1).

The role of miR-485-5p for suppressing growth and metastasis in different type of cancers have been widely described (Hu et al., 2018; Huang et al., 2018). Nevertheless, a recent study reported the capacity of circ-PTCH1, which targets miR-485-5p, for promoting invasion and metastasis in RCC, using both *in vitro* and *in vivo* models. The circ-PTCH1 is derived from the patched-1 (*PTCH1*) gene, which is a key component of the hedgehog signaling pathway. If a mutation causes an inactivation of *PTCH1*, it induces an aberrant activation of the hedgehog signaling pathway, which is related to initiation, proliferation, metastasis, and therapeutic resistance in different tumors (Mancuso et al., 2004; Hasanovic and Mus-Veteau, 2018). Moreover, circ-PTCH1 was found to be overexpressed in RCC tissues. In addition, adverse clinical phenotypes (advanced Fuhrman grade) have been positively correlated with circ-PTCH1 levels. Regarding the molecular mechanisms, circ-PTCH1 binds to miR-485-5p, thus stimulating migration and invasion of RCC, due to the increase of the expression of the matrix metallopeptidase-14 (*MMP14*) gene. Notably, the activation of the miR-485-5p/MMP14 axis through circ-PTCH1 leads to the activation of the EMT process (Liu et al., 2020).

Circ-CSNK1G3 is another circRNA with an oncogenic role in RCC, due to its involvement in RCC metastasis. Li et al. analyzed the expression of circ-CSNK1G3 in RCC tissues and observed overexpression when compared with healthy tissues. Furthermore, they also observed that it was able to promote tumor growth and metastasis in RCC, since circ-CSNK1G3 was able to increase miR-181b, which inhibits several tumor suppressor genes, such as *TIMP3*, *LATS2*, *NDRG2* and *CYLD*. A decreased *TIMP3* leads to an enhanced EMT process, which promotes cancer metastasis. In fact, the authors observed that when miR-181b was inhibited, the EMT-promoting effects caused by increased circ-CSNK1G3 were notably reversed, and overexpression of *TIMP3* was also able to reverse tumor growth. Of note is that EMT process activation as well as the VEGF signaling pathway contribute to tumor initiation (Fantozzi et al., 2014). circ-CSNK1G3 contributes to RCC growth and metastasis inducing miR-181b expression, which leads to a TIMP3-mediated EMT process (Li W. et al., 2021). Thus, circ-CSNK1G3 is another candidate that can be used as a diagnostic and prognostic biomarker.

Similarly, after analyzing a large number of circRNAs using microarray-based circRNA/gene expression profiling in RCC and normal tissues, Zhang et al. found circ-000926 as a key mediator of RCC cell growth, migration and invasion. In fact, the downregulation of circ-000926 resulted in the inhibition of these pathological processes, in addition to suppressing EMT and tumor growth. Mechanistically, circ-000926 indirectly increases *CDH2* expression through binding to miR-411, thus preventing the inhibition of *CDH2* by miR-411 (Zhang et al., 2019).

Another circRNA implicated in RCC carcinogenesis is circ-0035483, which was found to be upregulated in RCC tissues and cells. Liu et al. observed that when circ-0035483 was downregulated, proliferation, invasion, migration, EMT and glycolysis in RCC cells were inhibited. In addition, they also found that circ-0035483 targets miR-31-5p, which negatively regulates *HMGA1*, thus inhibiting the malignant behavior of RCC cells (Liu et al., 2021). Thus, the overexpression of circ-0035483 is related to tumor growth by regulating the miR-31-5p/HMGA1 axis.

Solute carrier family 39 member 14 (*SLC39A14*) is a SLC39A transmembrane metal transporter family member, which mediates the cellular uptake of zinc (Zn), iron (Fe) (Beker Aydemir et al., 2012; Jenkitkasemwong et al., 2015) and manganese (Mn) (Tuschl et al., 2016; Xin et al., 2017). *SLC39A14* gene mutations have been related to Mn^+2^ accumulation in the circulation and brain. *SLC39A14* deficiency impairs hepatic Mn^+2^ uptake and biliary excretion (Cotzias and Papavasiliou, 1964). Recently, the circ-001842/miR-502-5p/*SLC39A14* axis was described as being related to RCC. Zeng et al. observed an overexpression of circ-001842 in RCC tissues and its correlation with poor prognosis (*p*-value of 0.008). In addition, it was demonstrated that circ-001842 sponges miR-502-5p, which targets *SLC39A14*, and thus, RCC patients showed high levels of circ-001842 and *SLC39A14*. Furthermore, elevated expression levels of *SLC39A14* resulted in tumor growth *in vivo*, and increased RCC cell proliferation, migration and invasion, as well as EMT *in vitro* (Zeng et al., 2020). Due to the involvement of circ-001842 in controlling different pathways which cause tumor growth, it could be a good target for RCC treatment. In addition, due to the function of *SLC39A14*, it could be also a good biomarker for RCC prognosis.

Another interesting circRNA is circ-MYLK, which is notably up-regulated in RCC. Elevated circ-MYLK expression leads to an increase in tumor size, distant metastasis and poor prognosis in RCC patients. Furthermore, circ-MYLK silencing represses RCC growth and metastasis in both cell lines and patients. Mechanistically, circ-MYLK binds to miR-513a-5p, avoiding the deleterious role of miR-513a-5p in VEGF-C expression levels, thereby promoting the tumorigenesis of RCC cells (Li et al., 2020b). Likewise, the elevated levels of circ-MYLK found in RCC suggests circ-MYLK is an oncogenic circRNA, that could be used as a diagnostic and prognostic biomarker of RCC, as well as a therapeutic target in the treatment of kidney cancer, since the inhibition of circ-MYLK should increase the circulating levels of miR-513a-5p and therefore reduce the expression levels of VEGF-C, blocking cancer metastasis.

Similarly, circ-PRRC2A was involved in angiogenesis and RCC metastasis both *in vitro* (human derived ACHN and Caki-1 cells) and *in vivo* using mice models. In fact, the overexpression of circ-PRRC2A was reported to be positively associated with advanced clinical stage and worse survival in RCC patients. The authors postulated circ-PRRC2A as a good biomarker for the diagnosis and prognosis of RCC. Regarding the molecular mechanisms, circ-PRRC2A was able to prevent the degradation of tissue-specific oncogene *TRPM3*, through sponging miR-514a-5p and miR-6776-5p (Li W. et al., 2020). Furthermore, circ-PRRC2A promotes tumor EMT and the RCC aggressiveness.

In this regard, circ-TLK1 is a circRNA derived from the *TLK1* gene, which codifies for a cell cycle checkpoint protein involved in chromatin assembly. Interestingly, circ-TLK1 was found to be overexpressed in RCC tissue from patients and its expression correlated positively with distant metastasis and unfavorable prognosis. In addition, when circ-TLK1 was silenced, RCC cell proliferation, migration and invasion, were significantly inhibited both *in vitro* and *in vivo* (nude mice). Furthermore, it has been demonstrated that circ-TLK1 sponges miR-495-3p and increases proto-oncogene *CBL* expression, because it is the target of miR-495-3p, thus facilitating the proliferation and metastasis of RCC. In fact, Lei et al. (2021) also observed that when circ-TLK1 was downregulated, both the proliferation and metastasis were reduced and apoptosis was increased in RCC cells, by controlling the miR-495-3p/CBL axis. Furthermore, circ-TLK1 is also able to sponge miR-136-5p, thus promoting proliferation and metastasis of RCC. Moreover, Li et al. observed that circ-TLK1 was mainly in the cytoplasm and, through sponging miR-136-5p, it was able to positively regulate *CBX4* expression, which in turn was positively correlated with *VEGF-A* expression in RCC tissues. Likewise, *CBX4* knockdown inhibited *VEGF-A* expression in RCC cells (Li et al., 2020a). Thus, in conclusion, circ-TLK1 plays a critical role in RCC because of its ability to modulate different pathways related to RCC development.

### Circular RNAs Are Differentially Expressed in Males and Females With Kidney Cancer

Some authors have demonstrated that several molecular processes are activated differently in males and females during the development and progression of RCC, which is of special relevance for developing personalized therapies for this type of cancer. For instance, there are evidence relating the androgen receptor (AR) with RCC initiation and progression (DeVita et al., 2008; Chow et al., 2010; Chang et al., 2014; Zhu et al., 2014; Zhai et al., 2016; Zhai et al., 2017; Gong et al., 2021). Similarly, estrogen receptor beta (ERβ) has also been related to ccRCC progression (Han et al., 2018). Interestingly, gender affects the epidemiology of RCC, with a 1.5:1 ratio (men: women) (Capitanio et al., 2019). In fact, 114,000 men and 61,000 women died due to RCC in 2018 (Padala et al., 2020). Thus, gender may affect the molecular mechanisms implicated in RCC progression (Table 1).

Regarding with the positive link between the AR and RCC initiation and progression, Gong et al. described that AR enhances the transcription of *DHX9*, which induces the participation of a regulatory protein in circRNA biogenesis, producing the downregulation of circ-EXOC7. In addition, circ-EXOC7 sponges miR-149-3p, suppressing the expression of *CSF1* by binding to the 3′ UTR region of *CSF1* mRNA. Moreover, AR has also been positively correlated with miR-149-3p and negatively correlated with *CSF1* in AR-positive ccRCC tissues. In fact, preclinical studies developed in a mouse model also validated the suggestion that by altering the expression of circ-EXOC7 or AR, and therefore, targeting the AR/*DHX9*/circ-EXOC7/miR-149-3p/*CSF1* axis, metastasis is suppressed (Gong et al., 2021).

In a similar way, Han et al., demonstrated that circRNAs not only regulate the expression of key proteins during RCC development, but also proteins which in turn can regulate circRNAs, thus modulating tumor response. In this regard, these authors used a female mouse model and different culture cell lines demonstrating that the ERβ protein is able to suppress the expression of circ-ATP2B1 through the binding of ERβ to the 5′-promoter region of its host gene, the ATPase plasma membrane Ca^2+^ transporting 1 (*ATP2B1*). This inhibition induces an increase in the expression of fibronectin 1 (*FN1*) and can therefore enhance ccRCC cell invasion. In addition, a positive correlation was established between ERβ expression levels and the stage and prognosis of the disease (*p*-value of 0.001) (Han et al., 2018).

These findings open new avenues in the biomedical field of RCC and demonstrate the high bidirectional regulation that occurs between circRNAs and protein expression, commonly through intermediate mechanisms such as miRNAs, but sometimes, directly though the binding of circRNA to proteins. Obviously, more studies are needed to clarify the role that sexual hormones play in the development and migration of RCC.

### Circular RNAs Implicated in Kidney Cancer Apoptosis and Autophagy

The control of cell death mechanisms, such as apoptosis and autophagy, have been related to circRNAs as a cancer regulatory mechanism. Autophagy is a catabolic conserved cellular process for homeostasis maintenance, through damaged protein aggregates or organelles degradation under physiological and pathological conditions. In fact, in many types of human cancers, circRNAs have been related to cancer development and progression by activation or inhibition of autophagy and/or apoptosis (Du et al., 2017; Jin et al., 2018; Cui et al., 2021; Zhou et al., 2021) (Table 1).

In this regard*,* the Y-box binding protein-1 (*YBX1*) plays a role in cancer progression, having been described as being involved in the activation of MAPK/ERK (mitogen-activated protein kinase/extracellular regulated kinase) pathway (Zeng et al., 2021). Furthermore, the role of the MAPK/ERK pathway in tumorigenesis has been widely described, due to its involvement in many cellular processes, such as stress responses, apoptosis, cell proliferation and differentiation (Guo et al., 2020). Likewise, Zhao et al. described an overexpression of circ-SAR1A in RCC tissues, especially in patients with lymph node metastasis in advanced RCC Fuhrman grade. In addition, functional experiments performed *in vitro* demonstrated that circ-SAR1A suppression caused a decrease in cell growth and invasion, by circ-SAR1A sponging miR-382, which targets *YBX1* and reduces its expression, promoting RCC tumor growth and invasion. Therefore, circ-SAR1A suppression, causes an overexpression of miR-382, which blocks *YBX1* expression, thus preventing tumor expansion (Zhao et al., 2020). In addition, it has also been described that miR-382 inhibits tumor progression by targeting *SETD8* in other types of cancer (Chen et al., 2017). However, because *SETD8* codifies for a histone-lysine N-methyltransferase and it can also be expressed in RCC, it is feasible that promoting miR-382 expression through the inhibition of circ-SAR1A may be a potential treatment for RCC.

Remarkably, another circRNA, circ-NUP98, was proposed as a potential biomarker of RCC stage, due to its correlation with poor prognosis in patients with RCC. In fact, circ-NUP98 has been found to be overexpressed in RCC tissues and kidney-derived cell lines, such as ACHN, 786-O, Caki-1. In addition, when migration and invasion were repressed in RCC, the silencing of circ-NUP98 promoted caspase 3-dependent apoptosis. Mechanistically, circ-NUP98 sponges miR-567, which regulates *PRDX3* (Thioredoxin-dependent peroxide reductase 3) expression, a gene related to cell protection against oxidative stress through detoxifying peroxides (Cao et al., 2007) and also with NF-κβ activation in the cytosol, due to its role for acting synergistically with MAP3K13 (Masaki et al., 2003). Furthermore, the silencing of circ-NUP98 induced the overexpression of miR-567, which was related to the down-regulation of *PRDX3* expression. The role of miR-567 in cancer proliferation has previously been described by other authors, and its role in regulating cyclin dependent kinase-8 (CDK-8) was recently proposed as a mechanism for inhibiting cell proliferation and inducing apoptosis (Elkady et al., 2021). Likewise, miR-567 dysregulation was related to carcinogenesis in other cancers, such as breast cancer, favoring cell proliferation and migration (Bertoli et al., 2017). Furthermore, regarding RCC, some studies reported that silencing of circ-NUP98 reduced the expression of different proteins participating in the EMT pathway (Yu et al., 2020).

Similarly, circRNA-400068 is another interesting circRNA which has been related to RCC and, unlike the rest of circRNAs, it was found to be upregulated in plasma exosomes from RCC patients, as well as in tissue samples and cells. In this regard, functional analysis demonstrated that circ-400068 is able to bind to miR-210-5p, which in turn increases *SOCS1* levels, thus regulating cell proliferation and apoptosis (Xiao and Shi, 2020).

Yan *et al.* analyzed the role of circ-0035483 in gemcitabine sensitivity in RCC. The authors found that circ-0035483 was able to facilitate gemcitabine-induced autophagy and enhanced the resistance of RCC to gemcitabine in nude mice. Furthermore, by silencing circ-0035483 the sensitivity to gemcitabine increased *in vivo*. Mechanistically, circ_0035483 binds and therefore, inhibits, miR-335, which in turn results in increased levels of *CCNB1* and autophagy-related proteins (Yan et al., 2019). These findings postulate circ-0035483 as a feasible theragnosis biomarker for gemcitabine-related treatments.

## Clinical Significance of Circular RNAs in Renal Cell Carcinoma

Kidney cancer is one of the most prevalent cancers worldwide, with RCC being the deadliest urological cancer (Padala et al., 2020). Nonetheless, at 10 years of follow-up 62% of ccRCC patients remain alive (Leibovich et al., 2010). However, the survival rate varies depending on the stage at diagnosis, being from 93% when ccRCC is diagnosed at stage I to 12% when it is diagnosed at stage IV, which is metastatic cancer. Early diagnosis of kidney cancer is currently a challenge, mainly because of the absence of specific clinical symptoms (Howlander et al., 2019). Therefore, there is an urgent need to find new effective clinical biomarkers to help diagnose kidney cancer in early stages and avoid progression to metastatic stages, in which mortality exponentially increases. Moreover, new specific therapies will help reduce this high associated mortality.

For years, people with kidney cancer have had very few available treatments beyond surgery. However, over the past decade, there has been an explosion of therapies targeting kidney cancer, focused on blocking specific genes and proteins that control tumor growth (i.e., Temsirolimus or Everolimus) and the generation of new blood vessels (i.e., Sunitinib, Sorafenib or Pazopanib) (American Cancer Society, 2021). These drugs have demonstrated to be effective against advanced cancer but fail to stop tumor recurrence (Dorff et al., 2009). Similarly, while the use of another group of drugs that induce the immune system to attack the tumors is promising, it is very difficult to avoid the side effects associated with these new therapies (Corsello et al., 2013; Kroschinsky et al., 2017).

The detection of kidney cancer in the early stages would notably increase patient survival and increase the effectiveness of therapeutic strategies able to prevent the proliferation, migration and invasion of tumor and even undergo less aggressive therapies. However, the diagnosis of kidney cancer remains a challenge for clinicians and researchers worldwide, specially at early stages. In this scenario, circRNAs are considered to be promising non-coding RNAs for the diagnosis and prognosis of kidney cancer (Wang et al., 2020) and some authors have even proposed the possibility of developing anti-circRNA-based therapies, as well as circRNA-based therapies aimed at sponging miRNAs participating in kidney cancer development and progression (Bai et al., 2019; Bai et al., 2020).

CircRNAs modulate a wide range of molecular responses related to proliferation, migration, invasion, EMT activation, VEGF activation, apoptosis and autophagy, which are considered relevant biological processes that are altered in kidney cancer. In addition, circRNAs are both tissue-specific and developmental stage-specific molecules, making them promising biomarkers for differentiating among different stages of cancer (Di Agostino et al., 2020). Moreover, circRNAs have demonstrated its ability to predict postoperative recurrence in some cancers such as stage II/III colon cancer (Ju et al., 2019). These abilities of circRNAs for controlling key processes in RCC make them ideal candidates to be implemented in clinical settings, not only because they are feasible biomarkers, but also because of their ability to act theragnostic tools.

Many of the circRNAs described in this work may be useful not only for early RCC diagnosis but also for prognosis and treatment. In fact, some circRNAs are potential biomarkers such as circ-EGLN3 or circ-0001451, which showed an AUC of 0.98 and 0.704, respectively. Furthermore, circRNAs are promising therapeutic targets in RCC, since the use of anti-circRNA therapies for circRNAs which control tumor suppressor genes can control tumor growth (He et al., 2021). For example, as explained above, circ-CSNK1G3 increases the expression of miR-181b by blocking tumor suppressor genes. Therefore, anti-circRNA therapies focused on reducing circ-CSNK1G3 levels may modulate tumor growth. In addition, by promoting circRNAs, such as circ-MTO1, that can suppress tumor growth and metastasis, tumor growth can also be controlled.

## Conclusion and Future Perspectives

Kidney cancer pathophysiology is very complex, and understanding the molecular mechanisms that guide the different disease complications and the critical outcomes remains a prerequisite to find effective biomarkers and promising treatments to reduce the mortality, as well as the high number of morbidities that kidney cancer survivors suffer (Miller et al., 2008; Timsit et al., 2012).

The identification and characterization of circRNAs open new opportunities to understand kidney cancer at a molecular level. Consequently, circRNAs have become a very attractive field for future cancer research. However, at this moment, there is no information about circRNAs in papillary RCC, chromophobe RCC and collecting duct RCC. Therefore, it would be helpful to characterize the role of circRNAs controlling specific gene expression programs and regulating particular molecular pathways to better understand the underlying biology of those RCC histological subtypes (Yu and Kuo, 2019). In this regard, it is needed to anticipate the identification of RCC because it is usually diagnosed at a more advanced stage, which directly impacts in the disease-specific survival (Ljungberg et al., 2007). In addition, biomarkers are a current need to predict toxicity, tolerability, efficacy, and mechanisms of action of current used therapies. Moreover, biomarkers can be used to identify patients who can benefit from specific therapies. It is known that the better the biology of a cancer is understood, the better treatments can be developed, and this may also occur in RCC.

Importantly, kidney cancer is not a single nosological entity (Junker et al., 2013) and probably many different clinical forms will be described in the future. Genetic information together with circRNAs, miRNAs and other epigenetic mechanisms may provide relevant information and furnish light in the different clinical forms of kidney cancer. In these scenarios, circRNAs will become a very relevant molecules in the near future, specially playing a central role in diagnosis, prognosis, and theragnosis of kidney cancer, among others.
